# Anesthesiology trainees performing flexible scope intubation in spontaneously breathing patients in the left lateral position and the supine position: a prospective randomized trial

**DOI:** 10.1186/s12871-022-01636-2

**Published:** 2022-04-02

**Authors:** Poonyanuch Charoenkoop, Punchika Luetrakool, Tipanan Puttapornpattana, Nakkanan Sangdee

**Affiliations:** Department of Anesthesiology, Faculty of Medicine, Ramathibodi Hospital, Mahidol University, 270 Rama VI Road, Ratchatewi, Bangkok, 10400 Thailand

**Keywords:** Airway management, Flexible scope intubation, Flexible bronchoscope, Fiberoptic bronchoscope, Lateral intubation

## Abstract

**Background:**

Flexible scope intubation is an important airway management skill that requires hands-on training in a real airway. We compared flexible scope intubation by trainees between patients in the left lateral and supine positions.

**Methods:**

Forty patients aged 20 to 80 years with American Society of Anesthesiologists physical status class I to III were scheduled for elective surgery under general endotracheal anesthesia in Ramathibodi Hospital from February 2020 to June 2020. Patients were randomly assigned to be intubated in one of two positions: supine (Group S) or left lateral (Group L). Trainees performed flexible scope intubation in sedated patients under the supervision of an attending anesthesiologist. Intubation success, time to successful intubation, number of attempts, airway adjustment maneuvers, and hemodynamic changes were compared between groups.

**Results:**

Patient characteristics did not differ between groups except for Mallampati airway classification. The rate of successful intubation on the first attempt and intubation time did not significantly differ between groups. The proportion of patients who required a jaw thrust during intubation was significantly lower in Group L (10.5% vs. 85%; *P* < 0.01). Blood pressure and oxygen saturation declined in both groups after intubation. The relative risk of desaturation in patients in the left lateral position compared with the supine position was 0.44 (0.1649–1.1978).

**Conclusion:**

The rate of successful flexible scope intubation on the first attempt and intubation time did not differ between the groups. The proportion of patients who required a jaw thrust maneuver was significantly lower in patients in the left lateral position.

**Trial registration:**

https://www.thaiclinicaltrials.org/ (TCTR20200208001) on 08/02/2020.

## Introduction

The flexible bronchoscope has an important role in the management of difficult airways [1]. Newer scopes no longer use fiberoptic technology [2]. Flexible scope intubation (FSI) is a skill that anesthesiologists should have and be able to use when indicated [3]. Practicing this skill improves the likelihood of its successful implementation and reduces the incidence of complications [4]. Although beginners primarily practice FSI with mannequins, the rigid and static nature of the mannequin is a limitation. Trainees need hands-on experience in a real airway [1]. To simulate the difficult airway, the patient should be spontaneously ventilated after sedation is administered. Induction of general endotracheal anesthesia is a good opportunity to practice FSI. The major complications of FSI are hypoxemia from delayed intubation and soft tissue damage [5], which can lead to bleeding and/or airway edema. Unsuccessful FSI can cause complete airway obstruction and life-threatening oxygen desaturation, which are potentially catastrophic [6].

During esophagogastroduodenoscopy (EGD), the laryngopharyngeal structure can easily be observed during scope insertion [7]. Although the surgeon and anesthesiologist must share the airway during the procedure, the anesthetized patient can usually maintain a patent airway without the need for a jaw thrust maneuver [8]. Furthermore, the procedure is commonly performed with the patient in the left lateral decubitus position [9]. To approximate this situation, this study compared FSI performed by trainees on patients in the lateral and supine positions.

## Materials and methods

### Trial design

This prospective randomized study compared trainees performing FSI on patients in the lateral position with trainees performing FSI on patients in the supine position. It was conducted in a tertiary care hospital (Ramathibodi Hospital, Thailand) from February 2020 to June 2020. Ethics committee approval was obtained. All patients provided written informed consent.

### Patients

Patients aged between 20 and 80 years with American Society of Anesthesiologists (ASA) physical status class I to III who were scheduled to undergo elective surgery under general endotracheal anesthesia were eligible for study inclusion. Patients with ASA physical status classes IV and VI, a history of difficult intubation, poor cardiopulmonary reserve, coronary artery disease, cerebrovascular disease, or reactive airway disease were excluded. We also excluded patients who refused to participate or were anticipated to have a difficult intubation.

### Randomization

The 40 study patients were randomly assigned position of intubation (supine, Group S; and left lateral, Group L) by a data analyst on the day before surgery using a computer-generated random code (L or S) that was sent to the intubating anesthesiologist.

### Primary end points

The primary outcome was successful intubation, which was defined as intubation of the airway on the first attempt within 5 min. Timing started when the scope entered the oral airway and ended when the endotracheal tube was secured in the trachea, as confirmed by end-tidal carbon dioxide (ETCO_2_) capnography. The timing was reviewed by video recording.

### Secondary end points

Secondary outcomes included (1) time from insertion to vocal cord and total time; (2) number of attempts; and (3) measurements of heart rate, oxygen saturation, and systolic, diastolic, and mean arterial blood pressure (SBP, DBP and MAP, respectively), which were recorded before and within one minute after induction. Postintubation complications were examined within 24 h.

### Anesthesia procedures and quality control

On the day before surgery, all patients underwent a preoperative assessment, including airway examination and routine investigations. Consent was obtained. All patients fasted more than eight hours prior to intubation. After standard monitoring was initiated, glycopyrrolate 0.1–0.2 mg was injected intravenously, and 10% xylocaine spray was applied to the posterior pharynx. Patients were positioned for intubation in the left lateral or supine position as determined by randomization. The anesthetic trainee performed the intubation under supervision by an attending anesthesiologist. Patients were preoxygenated with 100% oxygen via facemask for at least three minutes prior to induction. Sedation with intravenous propofol 0.5–2 mg/kg and/or fentanyl 0.5–1 mcg/kg and/or midazolam 0.05–1 mg/kg was administered to maintain a Ramsay sedation scale score of 2 to 3.

First, the flexible intubating bronchoscope was introduced through the oral airway and then through the hypopharynx, vallecula, epiglottis and vocal cords. At the cords, 10 mg of 2% xylocaine was sprayed through the tip of the bronchoscope outlet. Then, the scope was advanced into the trachea, where another 10 mg of 2% xylocaine was sprayed. After the carina was identified, propofol 1–2 mg/kg was administered intravenously, and the endotracheal tube was railroaded during inspiration. The scope was then slowly withdrawn. After confirmation of tube placement in the trachea using ETCO_2_ capnography, nondepolarizing muscle relaxant (atracurium 0.5–0.6 mg/kg intravenous or cisatracurium 0.15–0.2 mg/kg intravenous) was administered. Intubation time was obtained from video recordings.

To maximize patient safety, anesthesiology trainees had to have practiced FSI with the mannequin in the supine position at least five times before intubating a patient. Intubation failure was defined as an intubation that required more than 10 min or more than two attempts. Rescue maneuvers included jaw thrust by an assistant when airway obstruction was noticed. Obstruction is described as contact between the uvula and the tongue or contact between the epiglottis and the posterior pharyngeal wall [10]. If oxygen saturation was less than 95% for more than 10 s, desaturation was recorded as a complication [11]; when this occurred, the trainee stopped FSI and applied 100% oxygen facemask ventilation. A second attempt could begin after appropriate desaturation treatment. If a second attempt was required or intubation required more than 10 min, the patient was immediately placed in the supine position for intubation by an attending anesthesiologist using conventional laryngoscopy.

### Sample size

Our sample size calculation was based on a previous FSI study [12]. Li et al. found that the success rate of first-attempt intubation was significantly higher in the lateral position group (97%) than in the supine position group (16%). We estimated that a sample size of at least 14 participants in each group was required to detect group differences with 80% power and assuming a 5% Type I error. A 20% dropout rate was estimated. Therefore, 40 participants (20 in each group) were enrolled.

### Statistical analysis

Statistical analysis was performed using SPSS software version 18.0 (IBM Corp., Armonk, NY, USA). Patient characteristics are reported as the means with standard deviations and ranges, medians with interquartile ranges, or frequencies with percentages. Intergroup differences were evaluated using Student’s t test for normally distributed continuous variables, the chi-square test for categorical variables, and the Mann–Whitney U test for nonnormally distributed continuous variables. *P* < 0.05 was considered significant.

## Results

### Study population

**Fig. 1:**
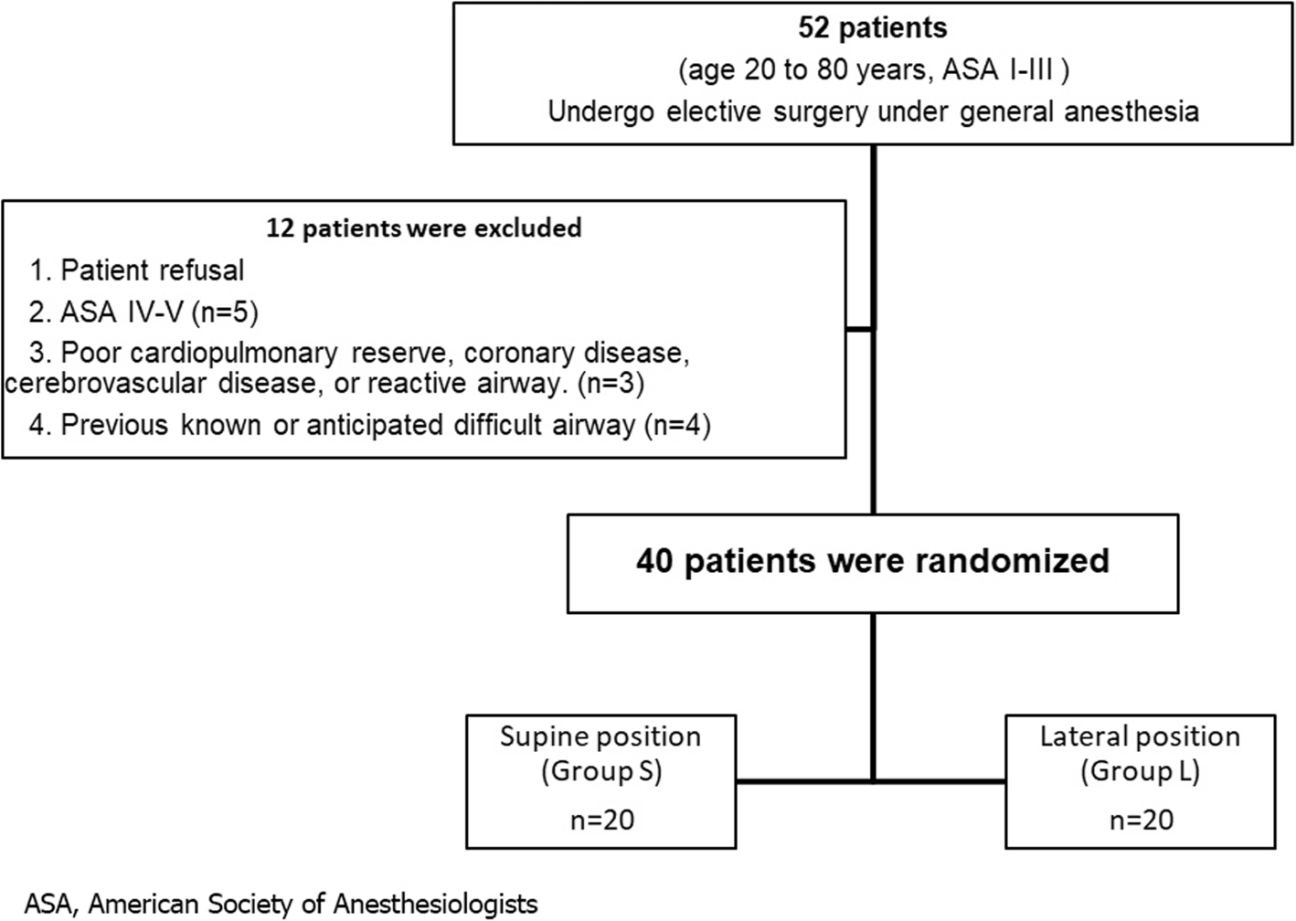
Consolidated Standards of Reporting Trials flow diagram

Fifty-two patients undergoing elective surgery under general endotracheal anesthesia were eligible for study inclusion. Among these, 12 patients did not meet the inclusion criteria. Therefore, 40 patients were enrolled and randomly assigned to intubation in the supine (Group S, *n* = 20) or left lateral position (Group L, *n* = 20) group. The study flow diagram is shown in the Consolidated Standards of Reporting Trials flow diagram (Fig. 1).

Overall, patient characteristics were well balanced between the groups (Table 1). Age, sex, weight, height, and body mass index did not significantly differ. Although Group L did not have any patients with a Mallampati class 3 or 4 airway, Group S had 9 (45%).

**Table 1:** Patient characteristics according to group

	**Group L** **(*n* = 20)**	**Group S** **(*n* = 20)**	**Statistic**	***P* value**
Sex, n (%)			0.114 ^‡^	0.736
Female	14 (70.0%)	13 (65.0%)		
Male	6 (30.0%)	7 (35.0%)		
Age (years), mean ± SD	54.30 ± 10.20	57.10 ± 8.53	-0.942 ^†^	0.352
ASA physical status class, n (%)			5.131 ^‡^	0.081
Class I	4 (20.0%)	2 (10.0%)		
Class II	12 (60.0%)	7 (35.0%)		
Class III	4 (20.0%)	11 (55.0%)		
**Physical examination**				
Height (cm.), mean ± SD	159.36 ± 9.91	158.88 ± 8.78	0.160 ^†^	0.873
Weight (kg.), mean ± SD	62.24 ± 12.06	63.22 ± 11.67	-0.261 ^†^	0.795
BMI (kg/m), mean ± SD	24.49 ± 4.23	25.59 ± 4.34	-0.779 ^†^	0.441
Mallampati class, n (%)			12.120 ^‡^	0.003*
Class 4	0	1 (5.0%)		
Class 3	0	8 (40.0%)		
Class 2	12 (60.0%)	6 (30.0%)		
Class 1	8 (40.0%)	5 (25.0%)		
Others, n (%)			6.754 ^‡^	0.123
Neck precaution	0	3 (15.0%)		
Thyromental distance 6 cm	1 (5.0%)	1 (5.0%)		
Mouth opening 3 cm	0	1 (5.0%)		
Mouth opening < 3 cm	0	1 (5.0%)		
Partial dentition	0	1 (5.0%)		
None	19 (95.0%)	13 (65.0%)		

*SD* Standard Deviation, *ASA* American Society of Anesthesiologists, *BMI* Body Mass Index

^*^P < 0.05, ^๏^ Z value, ^†^t value, ^‡^χ^2^ value

### Trainee experience

The level of training was not significantly different between the groups (*P* = 0.245). Group L included mostly third-year residents (50%), and Group S included mostly second-year residents (55%). Trainee experience prior to performing FSI was also not significantly different (*P* = 0.304) (Table 2). Some trainees commented that the anatomy was confusing with the patient in the left lateral position.

**Table 2:** Trainee experience in flexible scope intubation according to group

	**Group L** **(*n* = 20)**	**Group S** **(*n* = 20)**	**Statistic**	***P* value**
Level of training, n (%)			3.996 ^‡^	0.245
Fellow	0	1 (5.0%)		
Resident, first year	4 (20.0%)	2 (10.0%)		
Resident, second year	6 (30.0%)	11 (55.0%)		
Resident, third year	10 (50.0%)	6 (30.0%)		
Median number of experience(s), attempt(s) (range)	4 (0–9)	3 (0–6)	-1.028 ๏	0.304

^*^ *P* < 0.05, ^๏^ Z value, ^†^ t value, ^‡^ χ^2^ value

### Primary outcome

Almost all patients (95% in both groups) were successfully intubated by FSI. The rates of successful intubation in Groups L and S were 57.9% and 42.1%, respectively; the difference was not significant. The proportion of patients who required a jaw thrust during FSI was significantly higher in Group S (85% vs. 10.5%; *P* < 0.01) (Table 3).

**Table 3:** Intubation times, success rates, and proportions of patients who required a jaw thrust during flexible scope intubation according to group

	**Group L** **(*n* = 20)**	**Group S** **(*n* = 20)**	**Statistic**	***P*** value
**Intubation time**				
Identify vocal cord (seconds)	70.0 (35.0, 95.0)	60.0 (23.0, 65.0)	-0.760 ^๏^	0.447
Pass vocal cord (seconds)	130.0 (90.0, 194.0)	122.0 (75.0, 184.0)	-0.482 ^๏^	0.630
Pass ETT, identify ETCO_2_ (seconds) or total time (seconds)	209.37 ± 80.05	202.47 ± 91.48	0.247 ^†^	0.806
Success rate, % (n/N)				
All attempts	95 (19/20)	95 (19/20)	-	-
The first attempt	57.9 (11/19)	42.1(8/19)	0.947^‡^	0.330
The second attempt	42.1 (8/19)	57.9 (11/19)		
Jaw thrust, n (%)			21.631 ^‡^	< 0.001^*^
Yes	2 (10.5%)	17 (85.0%)		
No	17 (89.5%)	3 (15.0%)		

ETT Endotracheal tube, *ETCO*_*2*_, End-Tidal Carbon Dioxide

^*^ *P* < 0.05, ^๏^ Z value, ^†^ t value, ^‡^ χ^2^ value

### Secondary outcomes

The average total intubation time in Groups L and S was 209.37 ± 80.05 s and 202.47 ± 91.48 s, respectively (Table 4). The difference was not significant. Following successful intubation, all hemodynamic parameters (SBP, DBP, MAP, and heart rate) immediately responded in the same way in both groups. However, blood pressure (SBP, DBP and MAP) declined in both groups. Oxygen saturation also declined in both groups after intubation but remained above 95%. Desaturation below 95% occurred during intubation in four Group L patients and nine Group S patients. The relative risk of desaturation in patients in the left lateral position compared with patients in the supine position was 0.44 (0.1649–1.1978). No patient experienced harm.

**Table 4:** Vital sign measurements during flexible scope intubation according to group

Item	Group L (*n* = 20)	Group S (*n* = 20)	Statistic	P value
**Intubation data (vital signs)**				
**SBP (mm Hg)**, mean ± SD				
Baseline	136.40 ± 25.26	148.35 ± 22.27	-1.587 ^†^	0.121
After intubation within 1 min	104.20 ± 25.51	120.60 ± 28.25	-1.927 ^†^	0.062
Difference in SBP	-32.20 ± 23.92	-27.75 ± 20.20	-0.636 ^†^	0.529
**DBP (mm Hg)**, mean ± SD				
Baseline	80.80 ± 12.37	89.10 ± 15.51	-1.871^†^	0.069
After intubation within 1 min	66.05 ± 15.93	76.50 ± 18.67	-1.904 ^†^	0.064
Difference in DBP	-14.75 ± 12.23	-12.60 ± 19.36	-0.420 ^†^	0.677
**MAP (mm Hg)**, mean ± SD				
Baseline	94.85 ± 15.04	107.60 ± 15.96	-2.600 ^†^	0.013^*^
After intubation within 1 min	76.75 ± 16.49	90.35 ± 21.44	-2.249 ^†^	0.030^*^
Difference in MAP	-18.10 ± 15.65	-17.25 ± 21.19	-0.144 ^†^	0.886
**HR (beats/min)**, mean ± SD				
Baseline	82.65 ± 11.93	89.05 ± 18.50	-1.300 ^†^	0.203
After intubation within 1 min	83.80 ± 14.49	89.20 ± 14.94	-1.160 ^†^	0.253
Difference in HR	1.15 ± 8.87	0.15 ± 12.57	0.291 ^†^	0.773
**SpO**_**2**_** (%)**, mean ± SD				
Baseline	99.60 ± 1.10	100.0 ± 2.22	-0.721^†^	0.475
After intubation within 1 min	97.80 ± 3.47	98.95 ± 1.88	-1.303 ^†^	0.203
Difference in SpO_2_	-1.80 ± 3.49	-1.05 ± 2.74	-0.756 ^†^	0.454

*SD* Standard Deviation, *SBP* Systolic Blood Pressure, *DBP* Diastolic Blood pressure, *MAP* Mean Arterial Pressure, *HR* Heart Rate, *SpO*_*2*_ Oxygen Saturation

^*^ *P* < 0.05, ^๏^ Z value, ^†^ t value, ^‡^ χ^2^ value

## Discussion

This prospective randomized trial compared anesthesiology trainees performing FSI on patients in the supine position with trainees performing FSI on patients in the left lateral position. The rate of successful intubation on the first attempt and the time of intubation did not significantly differ between the positions. However, the proportion of patients who required a jaw thrust for airway assistance was significantly lower in the left lateral position group.

When practicing difficult airway intubation with a flexible scope, using sleeping patients who are breathing spontaneously provides two advantages. First, the flexible scope can be introduced into the airway without causing discomfort, and laryngospasm risk is minimized. Second, if airway obstruction develops or spontaneous ventilation is not maintained, the anesthetic drugs can be stopped immediately [1]. Induction of general endotracheal anesthesia provides a good opportunity for FSI practice. In a previous study [12], muscle relaxants were used, and the intubation time was limited to 120 s. In our study, the intubation time for nonparalyzed patients was expanded to within 10 min.

During EGD, sedated patients can maintain their airway without the need for a jaw thrust maneuver, and the vocal cords can usually be visualized [9]. Furthermore, the lateral or semilateral position provides a better glottis view without any assistance or need to displace the tongue [13]. Our study found that FSI in the left lateral position can be achieved mostly without performing the jaw thrust maneuver, but the side, left or right, might not affect the upper airway. In anesthetized patients in the supine position, the tongue or soft tissue of the throat can sag downward, which can obstruct the operator’s view [12]. The lateral position structurally improves maintenance of the passive pharyngeal airway and is associated with a lower degree of upper airway obstruction compared with the supine position [14, 15]. The jaw thrust maneuver can produce a significant sympathetic response [16], jaw pain [17], patient discomfort [18], and bruising [19]. It can also narrow the spinal canal [20], which may cause spinal cord injury in patients with cervical spine injury.

Our study demonstrated similar FSI success rates between patients in the left lateral and supine positions. The results are comparable to success rates after light wand-assisted intubation in the lateral decubitus position [21], video laryngoscope intubation in the left lateral position [22], and laryngeal mask intubation in the supine, right lateral, and left lateral positions [23].

In contrast, Li et al. [12] reported that intubation time was shorter in the lateral position group and that the first-attempt success rate was higher (97%) than that in the supine position group (16%). However, in their study, all patients were paralyzed, and FSI was performed by experienced anesthesiologists. Relaxation of muscle tone due to paralysis may have seriously affected their ability to intubate supine patients. Intubation in patients in the left lateral position is significantly effective in paralyzed patients but ineffective in nonparalyzed patients.

Moreover, some trainees in our study commented that they were unfamiliar with the view of the airway anatomy with the patients in the left lateral position. McCaul et al. reported deterioration of the laryngoscopic view in the left lateral position in 35% of patients [24]. Two trainees in our study were unable to complete FSI with the patient in either the supine or left lateral positions; multiple factors may be involved, including their skill and experience.

Intubation of patients in the left lateral position may have benefits. First, in patients undergoing surgery in the lateral position (e.g., endoscopic retrograde cholangiopancreatography), there is no need to reposition the patient after induction of general anesthesia, which may decrease the incidence of complications such as nerve injury, cervical spine injury, and accidental dislodgement of the endotracheal tube. Moving the patient may also cause loss of airway patency, change in endotracheal cuff pressure, tracheal mucosal damage, and microaspiration [25]. Second, this could be an alternative airway access in unavoidable circumstances, such as penetrating wounds at the back or cervical spine, in which the patient could not be positioned in the supine position [26].

Our study groups did not differ in patient characteristics except for Mallampati class. Group S had multiple patients with a class 3 or 4 Mallampati score. The Mallampati score is used to predict the ease of endotracheal intubation [27]. However, previous meta-analyses concluded that this score alone is inadequate for predicting difficult intubation [28]. Nonetheless, we found no difference in the success rate between the groups.

Overall, FSI in either position resulted in a transient decline in blood pressure. Because FSI causes less sympathetic stimulation than conventional direct laryngoscopic intubation [29] and propofol was administered to prevent the gag reflex prior to railroading of the endotracheal tube, FSI may require fewer doses of anesthetic drugs to attenuate the sympathetic response. Therefore, the incidence of drug side effects is expected to be lower.

To improve patient comfort and acceptance of FSI, sedation should be administered appropriately [30]. In moderate to deep sedation, respiratory depression and/or upper airway obstruction usually occur, which leads to hypoxemia. The reported incidence rates of hypoxemia during moderate to deep sedation range between 12 and 33% [31–34]. The incidence of oxygen desaturation in our study was 36.84%. To avoid any participant harm, we defined desaturation as oxygen saturation < 95% for > 10 s [11]. In other studies, it was defined as a saturation lower than 95% [31–34]. The incidence of desaturation in our study was lower in patients in the left lateral position; however, the difference was not significant. Future studies should include a greater number of participants to enhance statistical power and further examine the incidence of desaturation in sedated patients undergoing FSI in the left lateral position.

Some trainees in our study reported difficulty with performing FSI in patients in the left lateral position. The main reason for this appears to be unfamiliarity and confusion with the view, which can cause esophageal intubation. Practice is required to become proficient. All levels of trainees should occasionally practice FSI with patients in positions other than the traditional supine position.

## Conclusion

When performed by anesthesiology trainees in spontaneously breathing patients, the rate of successful FSI on the first attempt and intubation time did not differ between patients in the left lateral position and those in the supine position. The incidence of desaturation was lower in patients in the left lateral position. Furthermore, the proportion of patients who required a jaw thrust maneuver was significantly lower in patients in the left lateral position. However, trainees were more confused with the view, and esophageal intubation was more frequent in patients in the left lateral position. Anesthetic drugs should be carefully titrated during FSI induction to reduce hemodynamic fluctuations and drug side effects.

## Limitations

This study has several limitations. First, the examiners could not be blinded, as the intubation position was clearly visible. Second, the sample size is small. It was obtained from a previous study that had a significantly higher first‑attempt intubation success rate in the lateral position group [12], but their design was dissimilar, as described earlier. A larger sample size may provide a more reliable estimate of the success rate of lateral intubation.

## Data Availability

The datasets used and/or analyzed during the current study are available from the corresponding author upon reasonable request.

## Abbreviations

FSI: Flexible scope intubation
ETCO_2_: End-tidal carbon dioxide
SpO2: Oxygen saturation
